# Drug-encapsulated blend of PLGA-PEG microspheres: in vitro and in vivo study of the effects of localized/targeted drug delivery on the treatment of triple-negative breast cancer

**DOI:** 10.1038/s41598-020-71129-0

**Published:** 2020-08-25

**Authors:** S. M. Jusu, J. D. Obayemi, A. A. Salifu, C. C. Nwazojie, V. Uzonwanne, O. S. Odusanya, W. O. Soboyejo

**Affiliations:** 1 Department of Materials Science and Engineering, African University of Science and Technology, Km 10 Airport Road, Abuja, Nigeria; 2 Department of Mechanical Engineering, Worcester Polytechnic Institute, Worcester, MA 01609 USA; 3 Department of Biomedical Engineering, Gateway Park Life Sciences Center, Worcester Polytechnic Institute (WPI), 60 Prescott Street, Worcester, MA 01605 USA; 4 Department of Materials Science and Engineering, Worcester Polytechnic Institute, Worcester, MA 01609 USA; 5 Biotechnology and Genetic Engineering Advanced Laboratory, Sheda Science and Technology Complex (SHESTCO), Abuja, Nigeria

**Keywords:** Drug delivery, Materials science

## Abstract

Triple-negative breast cancer (TNBC) is more aggressive and difficult to treat using conventional bulk chemotherapy that is often associated with increased toxicity and side effects. In this study, we encapsulated targeted drugs [A bacteria-synthesized anticancer drug (prodigiosin) and paclitaxel] using single solvent evaporation technique with a blend of FDA-approved poly lactic-co-glycolic acid-polyethylene glycol (PLGA_PEG) polymer microspheres. These drugs were functionalized with Luteinizing Hormone-Releasing hormone (LHRH) ligands whose receptors are shown to overexpressed on surfaces of TNBC. The physicochemical, structural, morphological and thermal properties of the drug-loaded microspheres were then characterized using Fourier Transform Infrared Spectroscopy (FTIR), Scanning Electron Microscopy (SEM), Dynamic Light Scattering (DLS), Nuclear Magnetic Resonance Spectroscopy (NMR), Thermogravimetric Analysis (TGA) and Differential Scanning Calorimetry (DSC). Results obtained from in vitro kinetics drug release at human body temperature (37 °C) and hyperthermic temperatures (41 and 44 °C) reveal a non-Fickian sustained drug release that is well-characterized by Korsmeyer-Peppas model with thermodynamically non-spontaneous release of drug. Clearly, the in vitro and in vivo drug release from conjugated drug-loaded microspheres (PLGA-PEG_PGS-LHRH, PLGA-PEG_PTX-LHRH) is shown to result in greater reductions of cell/tissue viability in the treatment of TNBC. The in vivo animal studies also showed that all the drug-loaded PLGA-PEG microspheres for the localized and targeted treatment of TNBC did not caused any noticeable toxicity and thus significantly extended the survival of the treated mice post tumor resection. The implications of this work are discussed for developing targeted drug systems to treat and prevent local recurred triple negative breast tumors after surgical resection.

## Introduction

Cancer has been estimated to account for 9.6 million deaths in 2018^1^. It is the second leading cause of death next to cardiovascular disease which is the leading cause of death in the world^2^. The World Health Organization (WHO) has projected that over 13.1 million cancer-related deaths will occur by 2030, a time when cancer would likely overtake cardiovascular disease as the leading cause of death in humans^3^. Cancer can affect different parts of the body in both men and women. In the case of women, breast cancer, persists as the most commonly diagnosed cancer in women globally^1,4^. Notably, triple negative breast cancer (TNBC) is the leading cause of death of women, especially in low-resource countries^5,6^. TNBC is an aggressive and immunopathology subtype of breast cancer that usually does not respond to drugs that target ER, PR and HER2^7–10^ with relatively high mortality rate^7–9^. This is due to the poor prognosis that could result to late diagnosis, and ineffective treatment options^7–9^.

TNBC has been shown accounts for 15–20% of breast cancer cases in which there is a lack of estrogen receptors (ER), progesterone receptors (PR) and human epidermal growth factor receptor 2 (HER2)^8–10^. In such cases, bulk chemotherapy, radiation therapy and surgery (or their combination) are often used as triple negative breast cancer treatment strategies^10–12^. However, the side effects of treatments are often severe and cancer may also recur, triggering the need for revisiting therapies^13,14^.

Significant efforts have been made to develop drugs for the treatment of TNBC using platinum compounds^15^, EGFR inhibitors^16,17^, antiangiogenics therapy^18^, PARP inhibitors^19^, mammalian targets with rapamycin (mTOR)^20,21^, kinase inhibitors of SRC, Kit, and other kinases^18,22^. Recent efforts have also explored the use of implantable encapsulated drug systems^23^, drug-loaded/encapsulated microspheres^24,25^, nanospheres/nanoparticles^26,27^, liposomes^28^, dendrimers^29^, micelles^30^ and scaffolds^31^ for breast cancer treatment. Novel gold and magnetite nanocomposite heating systems have also been proposed for localized breast cancer treatment (via hyperthermia) and/or thermal ablation^27,32^. However, some of these approaches lack specificity and selectivity in their targeting of tumor cells.

Hence, in recent years, significant efforts have been made to develop targeted anti-cancer drugs for the improved treatment of cancer^33,34^. In most cases, targeted anti-cancer drugs can be engineered using specific Molecular Recognition Units (MRUs) or antibodies that interact specifically with receptors that are overexpressed on the surfaces of cancer cells^35–37^. Such specific targeting of cancer cells may, therefore, have the potential to reduce the potential side effects of cancer treatment. Since over 50% of human breast cancer cells express binding sites (receptors) for luteinizing hormone releasing hormone (LHRH), LHRH is one of the specific targeting receptors that can be used for the treatment of breast cancer^38–40^.

Polymeric materials are primary choices for controlled localized and targeted cancer drug delivery^41,42^. Polylactide-co-glycolide (PLGA) and polyethylene glycol (PEG) are FDA-approved polymers and have been widely explored for applications in drug delivery. This is due largely to their biocompatibility^43–45^. Furthermore, Poly (ethylene glycol) (PEG), a hydrophilic polymer, which decreases its interactions with blood components^44^. The proportion of poly lactic acid (PLA) and poly glycolic acid (PGA) in poly lactic acid co glycolic acid (PLGA) can also be used to control the degradation rates or drug release rates during controlled release from PLGA^43^.

Prior work has shown that the release of cancer drugs from biodegradable polymers can occur by diffusion^41,46–48^, solvent activation via osmosis or swelling of the system^46,49,50^, chemical or enzymatic reactions, or cleavage of the drug from the system^45,46,48,51^. Extended release over durations comparable to cancer treatment regimens is often a challenge. However, to the best of our knowledge, none of the prior studies have explored encapsulated new targeted cancer drug (PGS-LHRH) with FDA approved blend of polymer (PLGA and PEG)^52,53^. Furthermore, there has been no study that has explored the thermodynamics and kinetics of drug delivery from drug-loaded blends of polymer microspheres. Such studies are needed to provide insights into the thermodynamic driving forces and the release mechanisms that are associated with the release of targeted cancer drugs from microparticles for the localized treatment and prevention of recurred triple negative breast tumors after surgical resection.

This paper presents the results of an experimental study of a unique proportion of blend of polymers (PLGA and PEG) that were used to encapsulate targeted drugs (PGS-LHRH or PTX-LHRH) for the enhancement of sustained and localized delivery of targeted drugs for breast cancer treatment. This studies offer outstanding advantages that include targeted drug for controlled and prolong release period^54^. The in vitro drug release kinetics and thermodynamics with their degradation mechanisms were elucidated for micro-spherical drug-loaded polymer blends. Results from the drug release in vitro and in vivo experiment showed that the effect of targeted-loaded drug leads to decrease the viability of TNBC cells (MDA-MB-231). The induced cytotoxicity and changes in the underlying cytoskeletal structures of the MDA-MB-231 cells (that are associated with controlled cancer drug release) are also explored via Confocal Microscopy. The implications of the results are then discussed for the development of polymeric microspheres that are encapsulated with targeted cancer drugs. Such drugs are shown to have the potential for the controlled delivery of cancer drugs that prevent the regrowth or locoregional recurrence of TNBC after surgical resection.

## Results

### Microparticle characterization

**Figure 1:**
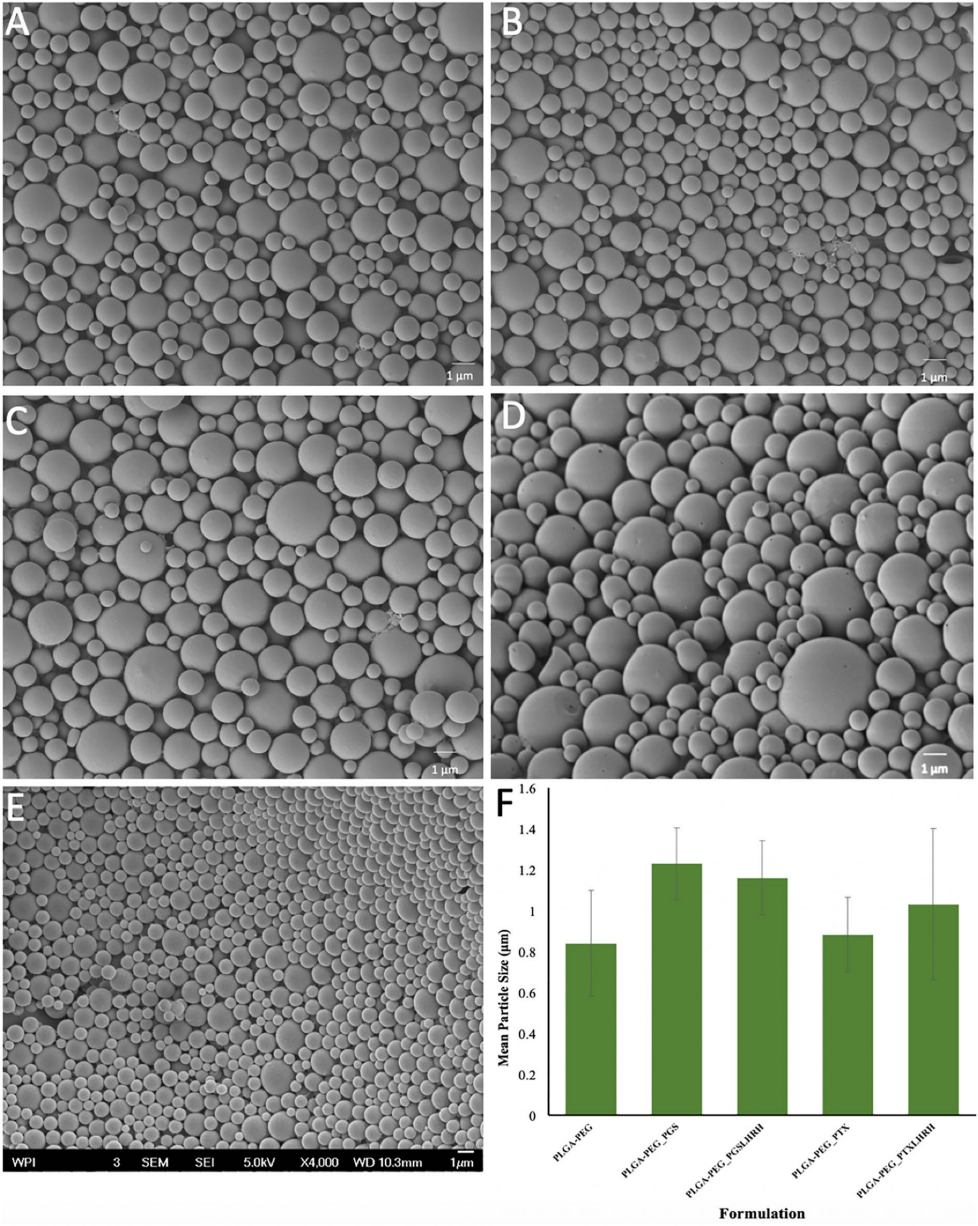
SEM images of (**A**) PLGA-PEG_PGS, (**B**) PLGA-PEG_PGS-LHRH, (**C**) PLGA-PEG_PTX, (**D**) PLGA-PEG_PTX-LHRH, (**E**) PLGA-PEG microspheres. (**F**) Mean particle size distributions of drug-loaded and control PLGA-PEG microspheres.

SEM images of the polymer blend drug-loaded microspheres with their and control microspheres are presented in Fig. 1A–E. Our results show that there are no significant morphological differences between the drug-loaded PLGA-PEG microspheres and the control PLGA-PEG microspheres. This suggests that the presence of drug did not significantly affect the morphologies of the drug-loaded microspheres. Furthermore, the mean particle sizes of the microparticles were between 0.84 and 1.23 μm (Fig. 1F). The hydrodynamic diameter obtained from the DLS (Table 1) were greater than the mean diameter obtained from the SEM (Fig. 1F). This could be attributed to the PEG being soluble in the DLS medium leading to a swollen structure with high water content^55^.

**Table 1:** The mean diameter (SEM), the hydrodynamic hydrometer (DLS) and the polydispersity index (PDI) values for the various PLGA-PEG microspheres formulations.

Formulation	SEM (μm)	DLS (μm)	PDI
PLGA-PEG	0.80 ± 0.26	3.14 ± 0.09	0.82
PLGA-PEG_PGS	1.23 ± 0.18	5.44 ± 0.23	0.67
PLGA-PEG_ PGS-LHRH	1.16 ± 0.18	6.92 ± 0.44	0.47
PLGA-PEG_PTX	0.88 ± 0.18	5.26 ± 0.53	0.58
PLGA-PEG_PTX-LHRH	1.03 ± 0.37	6.02 ± 0.80	0.39

**Figure 2:**
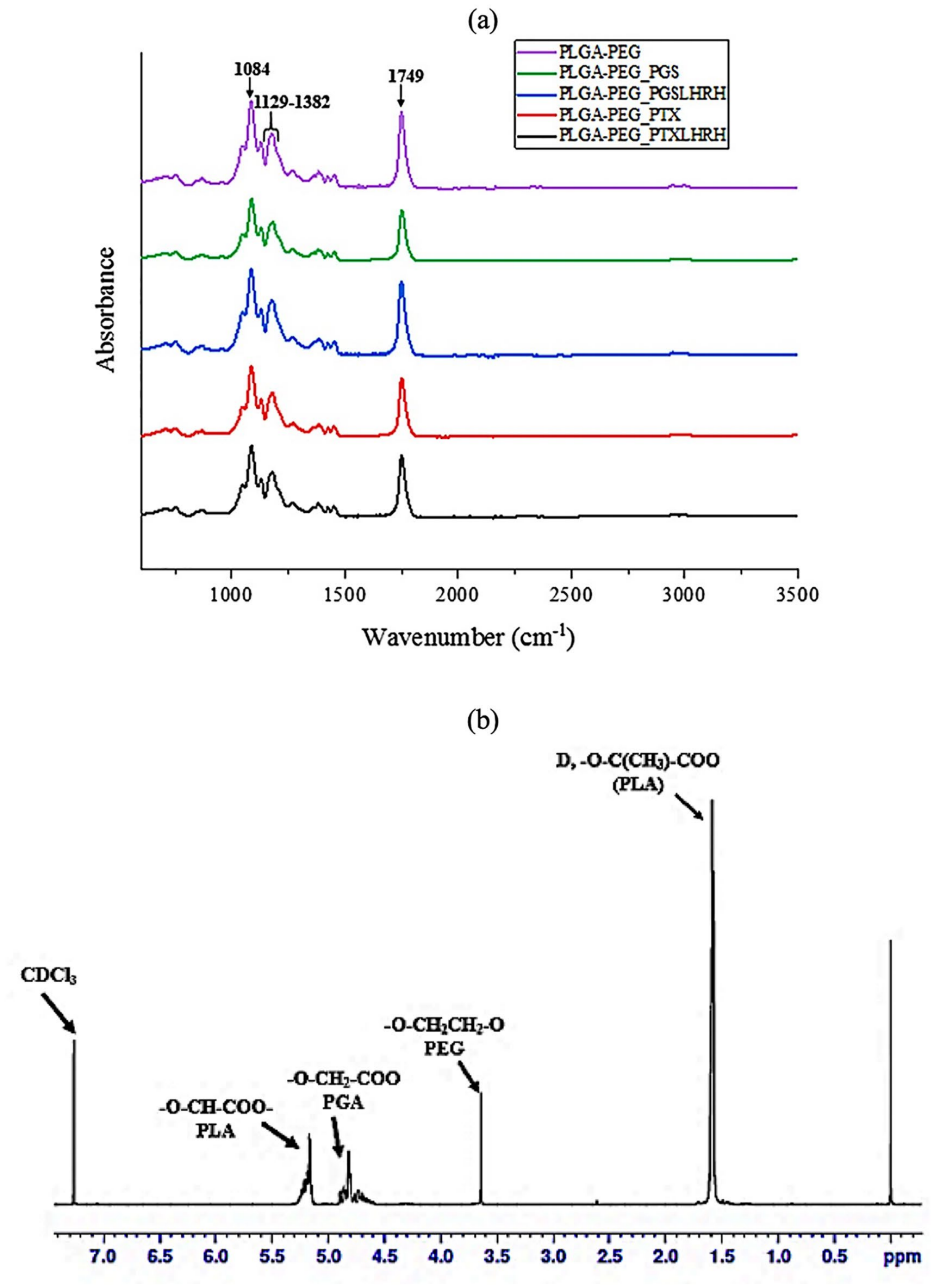
(**a**) FTIR spectra of the synthesized drug-loaded (PLGA-PEG_PGS, PLGA-PEG_PGS-LHRH, PLGA-PEG_PTX, PLGA-PEG_PTX-LHRH) microspheres and control (PLGA-PEG) microspheres. (**b**) A representative 1HNMR spectrum for drug-loaded PLGA-PEG microspheres.

The FTIR spectra obtained for the drug-loaded PLGA-PEG microspheres were similar to those of the control PLGA-PEG microspheres (Fig. 2a). This indicates that there was no significant modification on the chemical groups of PLGA and PEG due to drug loading. Hence, in each case, the characteristic peaks that were obtained for PLGA and the PEG polymer. These were present before and after drug loading. Thus, the FTIR spectra obtained for the drug-loaded and control PLGA-PEG microspheres showed a strong band at 1749 cm^−1^. This corresponds to the C=O stretch in the lactide and glycolide structure^53,56–58^. A characteristic peak of PEG was revealed at 1,084 cm^−1^. This is equivalent to the C-O stretch^56,59,60^. Clearly, the identical FTIR spectra of the drug-loaded microspheres (PLGA-PEG_PGS, PLGA-PEG_PGSLHRH, PLGA-PEG_PTX and PLGA-PEG-PTXLHRH) correspond to those of the spectrum of the blend of polymer (PLGA-PEG). Results from the drug-loaded spectra show the absence of characteristic intense bands of the drugs used (PTX, PG, PTXLHRH or PGSLHRH). In each case, the absence of the peaks may have been masked by the bands produced by the blend of polymer^61^. This result suggests the presence of drugs as a molecular dispersion in the blend polymer matrix due to the absence of chemical interaction between the blend of polymer (PLGA-PEG).

Similar HNMR spectra were obtained for all of the PLGA-PEG microsphere formulations, with four sets of principal peaks (ppm). Figure 2b shows representative HNMR spectra for the different formulations of PLGA-PEG microspheres. The peak at 3.64 ppm corresponds to the hydrogen atoms in the methylene groups of the PEG moiety^52,62^. Hydrogen atoms in the methyl groups of the d- and l-lactic acid repeat units resonated at 1.57 ppm with an overlapping pair^62,63^. A highly complex peak, due to several different glycolic acid, d-lactic, l-lactic sequences in the polymer backbone, was observed at 4.81 ppm and 5.20 ppm. This corresponds to the glycolic acid CH_2_ and the lactic acid CH, respectively^62^. Deuterated chloroform was used as a solvent and a chemical shit was seen at 7.26 ppm. These results suggest that the blend of polymers did not undergo chemical modification during drug loading and encapsulation.

**Figure 3:**
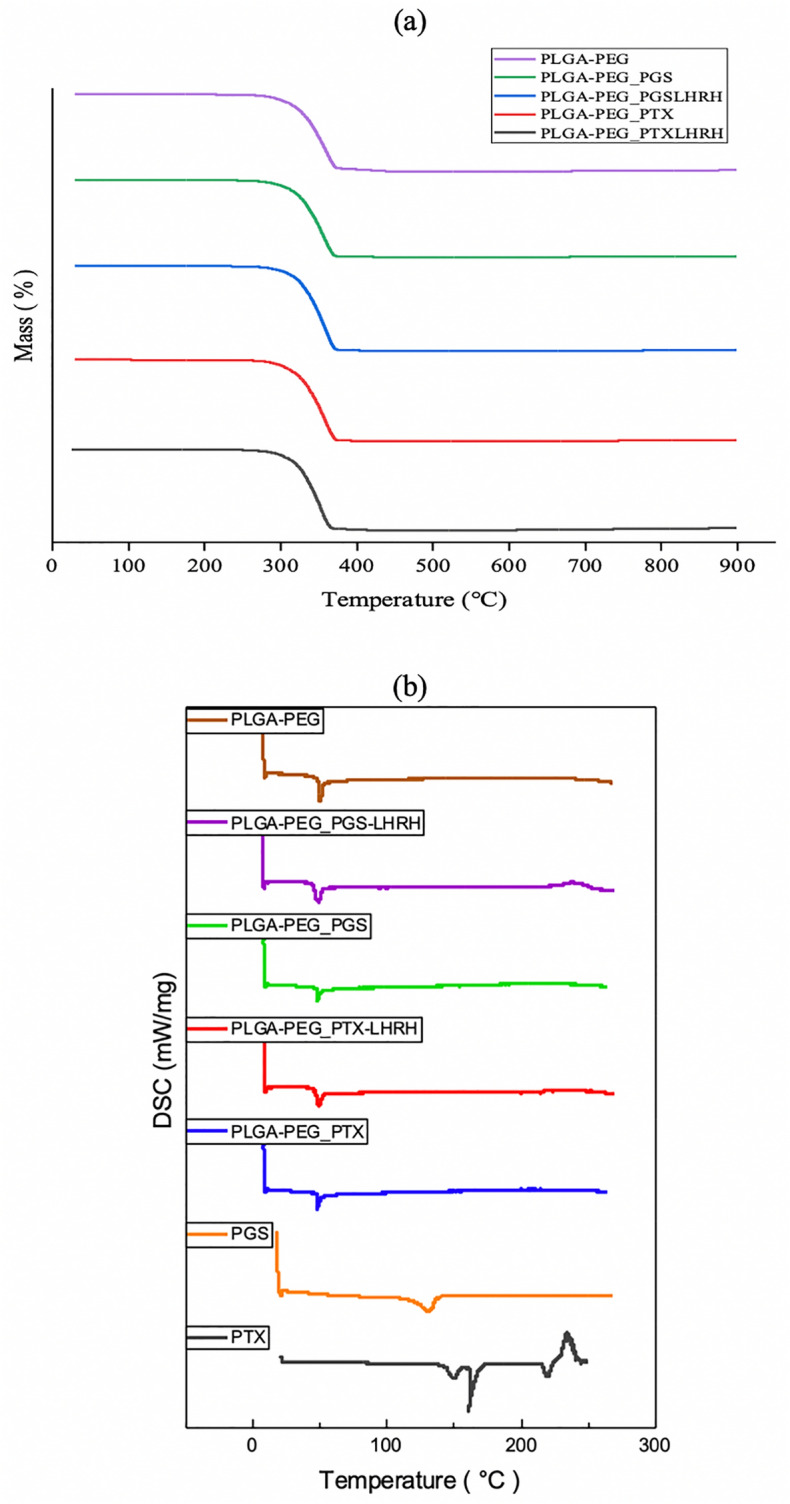
(**a**) TGA curves of control PLGA-PEG microspheres and drug-loaded PLGA-PEG microspheres. (**b**) DSC thermographs of freeze-dried drug-loaded and control PLGA-PEG microspheres, respectively.

Figure 3a below, shows the thermal decomposition process of control PLGA-PEG microspheres and drug-loaded PLGA-PEG microspheres obtained via Thermogravimetric Analysis (TGA). The TGA thermograms reveal one stage of weight loss. This suggests that the polymers and respective drugs mix but do not interact. The one step decomposition in the TGA analysis (Fig. 3a) may be due to the decomposition of the PLGA moiety in the blend^64^. The decomposition temperatures of the control PLGA-PEG microspheres and the drug-loaded PLGA-PEG microspheres are presented in Fig. 3b. The results show that the decomposition temperature decreases with drug loading.

The DSC thermograms are presented in Fig. 3b. This reveals that the control PLGA-PEG microspheres and drug-loaded PLGA-PEG microspheres exhibited similar endothermic events with a single defined peak. This suggests that the drug-loading did not affect the polymer structure. In the case of the control PLGA-PEG microspheres, the glass transition temperature (T_g_) and the melting temperature (T_m_) were measured to be 48.3 °C and 51.3 °C, respectively (Table 2). The ∆Cp corresponds to 0.411 J/(g K). However, in the case of drug-loaded PLGA-PEG microspheres, the T_g_ and T_m_ were lower than those of the control PLGA-PEG microspheres, leading to higher ∆Cp values. These changes in the measured values are attributed to the effects of the respective drugs, which act as a plasticizers for the polymer (PLGA)^65^.

**Table 2:** The Glass transition temperature (Tg), Endothermic peak and Delta Heat Capacity (∆Cp) values for the various PLGA-PEG microspheres formulations.

Drug-loaded composition	Glass transition temperature (Tg) (°C)	Endothermic peak (°C)	Delta heat capacity (∆Cp) J/(g K)	Decomposition temperature (°C)
PLGA-PEG	48.3	51.3	0.411	334.4
PLGA-PEG_PGS	47.0	49.3	0.635	327.2
PLGA-PEG_PGS-LHRH	47.8	50.2	0.497	322.3
PLGA-PEG_PTX	47.3	49.6	0.495	330.5
PLGA-PEG_PTX-LHRH	47.6	50.1	0.479	325.7

Furthermore, it was also observed that crystalline PTX had an endothermic peak corresponding to a melting point of 220 °C. Similarly, in the case of case of PGS, an endothermic peak was observed at 132 °C. It should be noted that due to the concentration and the very low drug loading of the drug in the respective microspheres, there was no any noticeable signature peaks of corresponding drug formed in each drug-loaded system. This result indicate that each drug encapsulated did not crystallize in the blend of polymer microspheres^66^. Generally, it was observed that the encapsulation of drug into the polymer microspheres did not significantly change the thermal properties of the drug-loaded polymer systems.

### In vitro drug release

**Figure 4:**
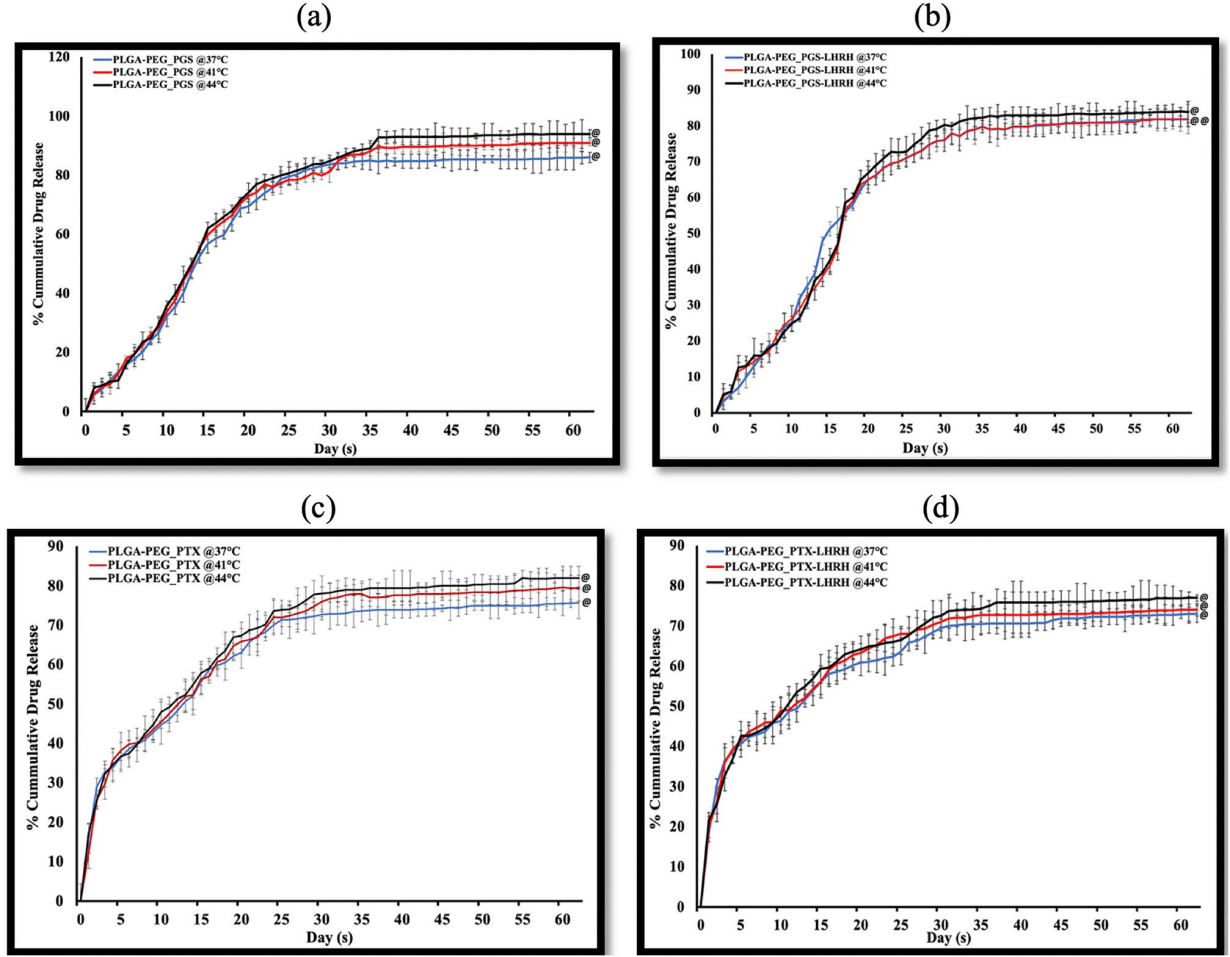
In vitro release profile of (**a**) PLGA-PEG-PGS microspheres, (**b**) PLGA-PEG-PTX, (**c**) PLGA-PEG-PGS-LHRH, (**d**) PLGA-PEG-PTX-LHRH drug-loaded microspheres at 37 °C, 41 °C and 44 °C, respectively. In all cases (n = 3, ^@^p > 0.05 vs. control).

Figures 4a–d show the time dependence of the percentage of cumulative drug release from the drug-loaded PLGA-PEG microspheres. All of the drug-loaded formulations revealed similar release profiles. However, the initial burst release after 48 h was strongly affected by the type of drug that was encapsulated. In the case of PLGA-PEG microspheres that were loaded with PGS or PGS-LHRH, lower levels of burst release were observed, compared to those obtained from PLGA-PEG microspheres that were loaded with PTX or PTX-LHRH. This was attributed to the hydrophilic and hydrophobic moieties in the PGS-based drugs^31^.

After 62 days, ~ 80% of PTX and PTX-LHRH drugs was released, while ~ 85% of PGS and PGS-LHRH was released over the same period. The slight decrease in the percentage of cumulative drug release from the PTX and PTX-LHRH drug is attributed to the stronger hydrophobic domain of PTX-based drugs. Finally, in this section, it is important to note that controlled release occurred from the microspheres (with ~ 60% release) within ~ 40 days. The respective drug encapsulation efficiencies and their drug loading efficiency obtained for the drug-loaded PLGA-PEG_PGS, PLGA-PEG_PGS-LHRH, PLGA-PEG_PTX, PLGA-PEG_PTX-LHRH, were determined to be ~ 46%, 40%, 72%, 38% and 12.3%, 14.2%, 16.1%, 9.8%, respectively. In each case of the drug release studies, the results were not significant since the p value for each drug at different temperatures considered are greater than 0.05. This implies that there was no significant difference when we use different temperatures. However, comparing the respective cumulative drug release, the results were considered to be significant with a p value < 0.05.

### Drug release kinetics

The drug release kinetics (Table 3) obtained from the drug release data that were fitted in the kinetic models [zero order ($$Q_{t} = Q_{O} + K_{0} \cdot t$$), first order $$\left( {\log Q_{t} = \log Q_{0} + {\raise0.7ex\hbox{${Kt}$} \!\mathord{\left/ {\vphantom {{Kt} {2.303}}}\right.\kern-\nulldelimiterspace} \!\lower0.7ex\hbox{${2.303}$}}} \right)$$, Higuchi model ($$Q_{t} = K_{H} \cdot t^{{{\raise0.7ex\hbox{$1$} \!\mathord{\left/ {\vphantom {1 2}}\right.\kern-\nulldelimiterspace} \!\lower0.7ex\hbox{$2$}}}}$$) and Korsmeyer–Peppas model $$\left( {\frac{{M_{t} }}{{M_{\infty } }} = Kt^{n} } \right)$$] showed clearly that the Korsmeyer–Peppas model provided the best fit to the experimental data obtained for the different drug-loaded PLGA-PEG microsphere formulations. In some cases, the release exponent ‘n’ was between 0.446 and 0.889, which is consistent with drug release by anomalous transport or non-Fickian diffusion that involves two phenomena: drug diffusion and relaxation of the polymer matrix^67^.

**Table 3:** The kinetic constant (K), correlation coefficient (R^2^) and Release exponent (n) of kinetic data analysis of drug released from the various PLGA-PEG microspheres formulations.

Formulations	Temperature (°C)	Zero order		First order		Higuchi model		Koresmeyer–Peppas		
		K	R^2^	K	R^2^	K	R^2^	K	R^2^	n
PLGA-PEG_PGS	37	1.232	0.711	0.014	0.505	12.786	0.857	1.920	0.973	0.870
PLGA-PEG_PGS-LHRH		1.226	0.749	0.015	0.529	12.578	0.882	1.837	0.977	0.889
PLGA-PEG_PTX		0.769	0.692	0.008	0.330	8.137	0.867	3.271	0.962	0.459
PLGA-PEG_PTX-LHRH		0.680	0.704	0.007	0.294	7.802	0.845	3.340	0.848	0.490
PLGA-PEG_PGS	41	1.315	0.752	0.014	0.513	13.533	0.875	1.982	0.969	0.855
PLGA-PEG_PGS-LHRH		1.236	0.760	0.015	0.548	12.607	0.884	1.785	0.976	0.885
PLGA-PEG_PTX		0.853	0.718	0.009	0.354	8.964	0.886	3.398	0.969	0.447
PLGA-PEG_PTX-LHRH		0.685	0.672	0.007	0.288	7.316	0.856	3.431	0.912	0.446
PLGA-PEG_PGS	44	1.371	0.757	0.014	0.519	13.987	0.867	2.034	0.953	0.842
PLGA-PEG_PGS-LHRH		1.281	0.749	0.015	0.551	13.085	0.873	1.809	0.966	0.881
PLGA-PEG_PTX		0.881	0.728	0.009	0.357	9.224	0.951	3.210	0.985	0.490
PLGA-PEG_PTX-LHRH		0.753	0.712	0.008	0.311	7.939	0.885	3.302	0.968	0.450

### Thermodynamics of drug release

**Figure 5:**
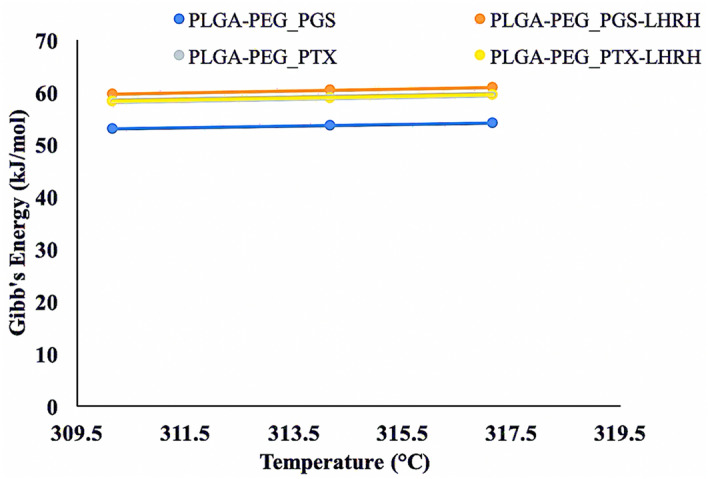
A plot of Gibb’s free energy versus Temperature for various drug-loaded PLGA-PEG formulations.

The thermodynamic parameters (Δ*G*, Δ*H*, Δ*S* and *E*_a_*)* that were obtained from this study are presented in Table 4. The change in the Gibb’s free energy (Δ*G)* was negative for all of the PLGA-PEG microsphere formulations. This indicates the feasibility and non-spontaneous nature of the drug release from the PLGA-PEG microspheres at all temperatures. Figure 5 shows a plot of Gibb’s free energy versus Temperature for various PLGA-PEG formulations. The negative values obtained for the change in entropy (Δ*S)* also confirm that there is a decrease in the disorder associated with drug release from the various PLGA-PEG microspheres. Furthermore, the positive values obtained for the change in enthalpy (Δ*H*) confirm that the drug release process (from all of the PLGA-PEG microspheres formulations containing PGS, PGS-LHRH or PTX-LHRH respectively) was endothermic. However, a positive *E*_a_ was obtained for the drug release from all the PLGA-PEG formulations, indicating that in all cases, the rate of drug release increased with increasing temperature.

**Table 4:** Thermodynamic parameters for the various PLGA-PEG microspheres.

Formulations	Temperature (°C/K)	E_a_ (kJ mol^−1^)	ΔS (kJ mol^−1^ K^−1^)	ΔH (kJ mol^−1^)	Δ*G* (kJ mol^−1^)
PLGA-PEG_PGS	37/310.15	6.720	− 0.170	6.720	52.871
	41/314.15				53.553
	44/317.15				54.064
PLGA-PEG_PGS-LHRH	37/310.15	4.379	− 0.178	4.379	59.586
	41/314.15				60.298
	44/317.15				60.832
PLGA-PEG_PTX	37/310.15	7.714	− 0.163	7.714	58.268
	41/314.15				58.920
	44/317.15				59.409
PLGA-PEG_PTX-LHRH	37/310.15	5.444	− 0.170	5.444	58.170
	41/314.15				58.850
	44/317.15				59.360

### Degradation of drug-loaded microspheres

**Figure 6:**
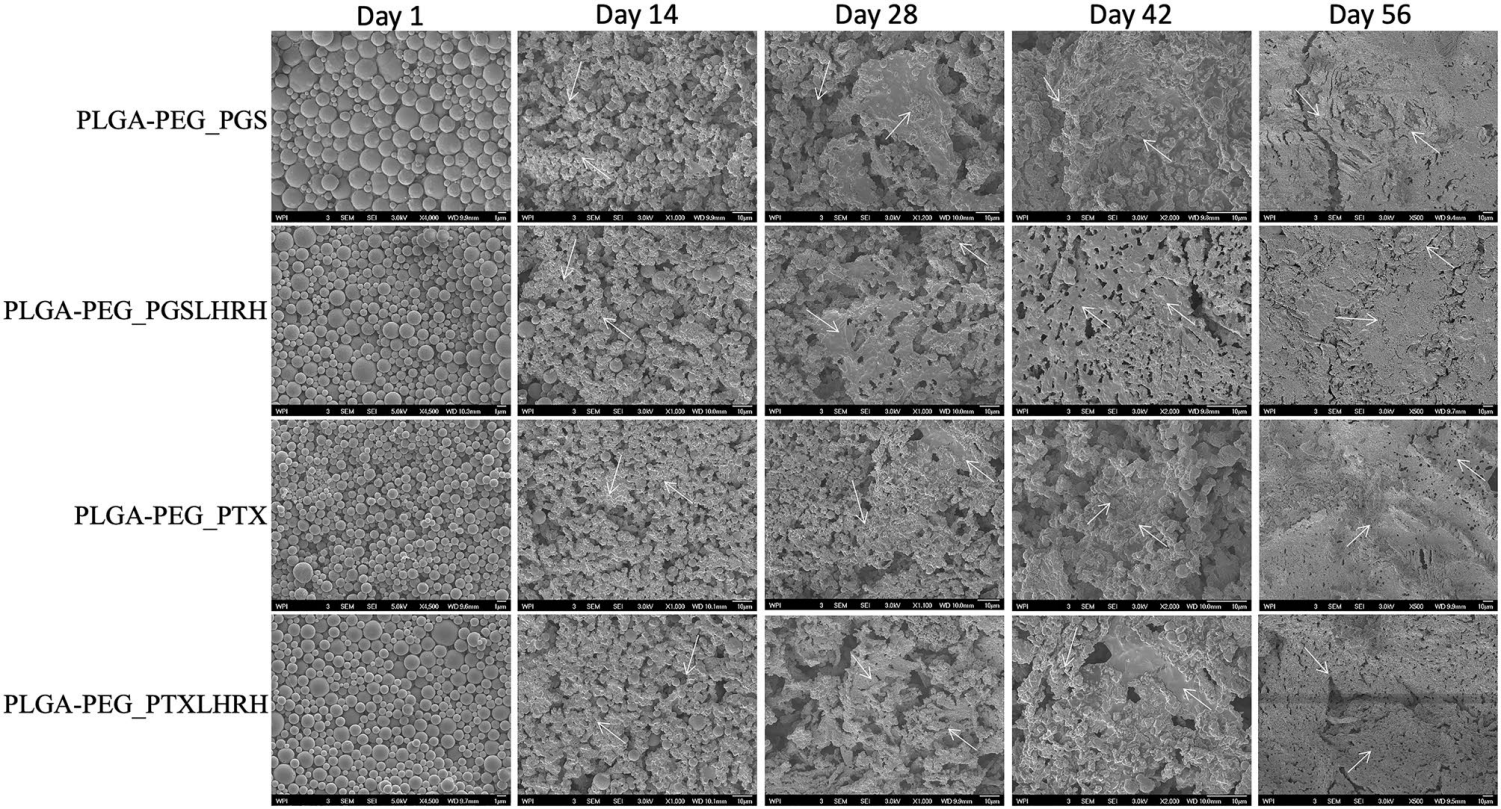
SEM images of surfaces of drug-loaded PLGA-PEG microspheres after 57 days exposure to phosphate buffer saline at pH 7.4: and cross-sections (note the different magnifications/scaling bars). The white arrows show evidence of the progression of material removal and degradation site.

SEM images of the degradation of the drug-loaded microspheres are presented in Fig. 6. Gradual morphological changes were observed within the 56-day period of the drug release experiments. After 24 h of exposure to the release medium (PBS, pH 7.4), the surfaces of the drug-loaded PLGA-PEG microspheres were still smooth with micropores. However, by day 14, morphological changes were observed. These included microsphere agglomeration, distinct micropores and less spherical shapes. Evidence of microsphere agglomeration and void formation was observed by Day 28. After 42 days of drug elution, the surfaces of the PLGA-PEG microspheres were completely eroded visibly larger pores. Further evidence of material removal was also observed after 56 days of drug elution, which was found to result in more porous structures than those that were observed before drug elution. The increased erosion is attributed to the hydrolytic degradation of the ester and drug leaching^68^.

### Cell culture

#### In vitro cell viability and drug cytotoxicity

**Figure 7:**
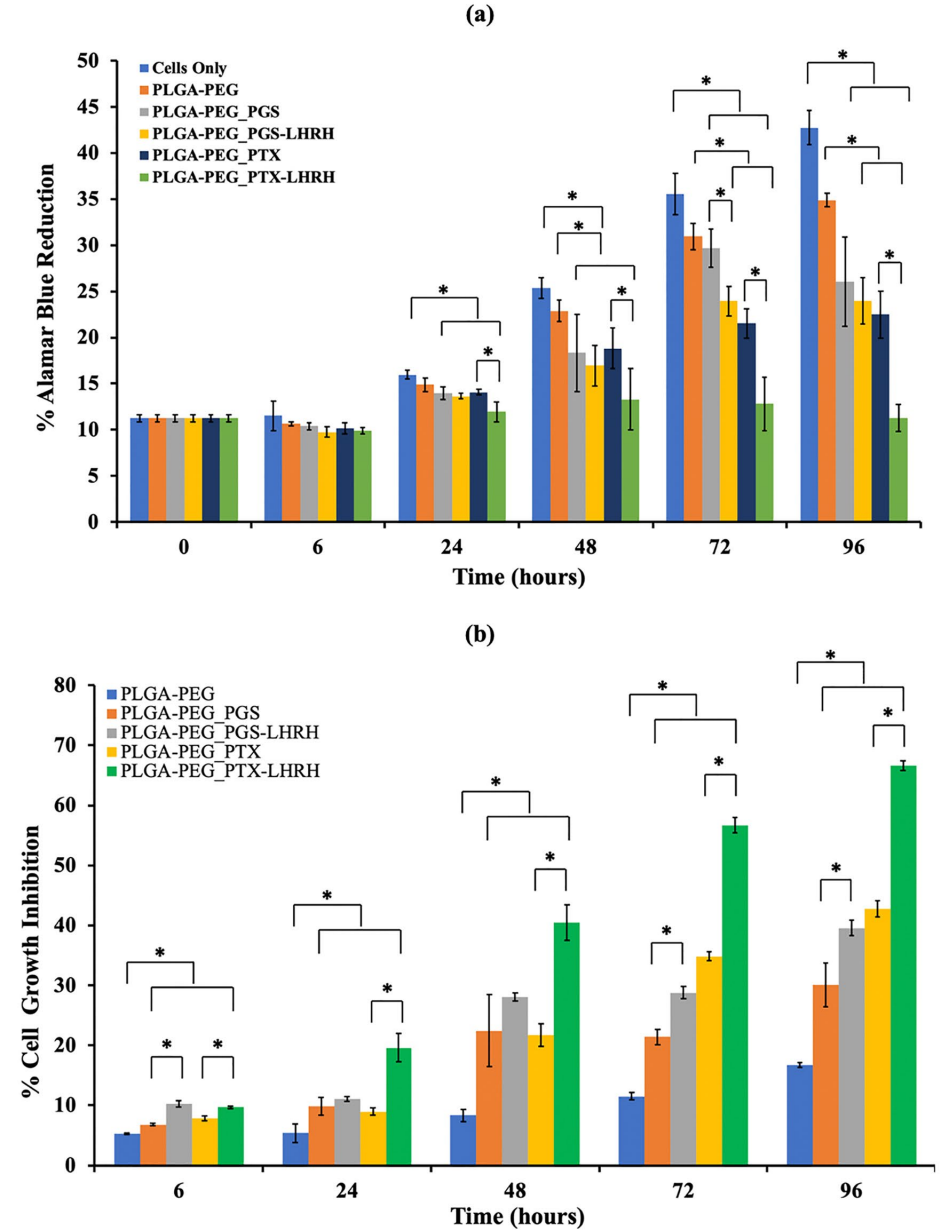
(**a**) Percentage alamar blue reduction for cells only (MDA-MB-231 cells), drug-loaded and control PLGA-PEG microspheres after 6, 24, 48, 72 and 96 h post-treatment. (**b**) Percentage cell growth inhibition for drug-loaded and control PLGA-PEG microspheres after 6, 24, 48, 72 and 96 h’ post-treatment [*p < 0.05 (n = 4)].

Figure 7a,b compares the percentage alamar blue reduction and percentage cell growth inhibition, respectively, for cells only (MDA-MB-231 cells), drug-loaded and control PLGA-PEG microspheres 6, 24, 48, 72 and 96 h post-treatment. The percentage alamar blue reduction measures the cell metabolic activity, which is a function of the cell viability and cell population. This implies that a higher percentage of alamar blue reduction value corresponds to a higher cell growth and, by extension, a higher cell viability. A two-way ANOVA with post hoc Tukey HSD multiple comparisons tests showed that, generally, the cell viability was significantly lower (p < 0.05) for the cells treated with drug-loaded PLGA-PEG microspheres than cells that were not exposed to drug elution from microspheres. Furthermore, the cells treated with PLGA-PEG microspheres loaded with conjugated drugs (PGS-LHRH, PTX-LHRH) were less viable than their counterparts that were loaded with unconjugated drugs (PGS, PTX). This means that the conjugated drugs were more effective at reducing the metabolic activities of the MDA-MB-231 cells than their unconjugated counterparts. The statistically significant group pairs of interest (p < 0.05) are highlighted in Fig. 7.

There was a slight reduction in cell viability when the cells were exposed to the control PLGA-PEG microspheres (no drugs), attributed to the cytotoxic effects of leached residual DCM solvent that was used to process the microspheres. However, the reduction in cell viabilities (Fig. 7a) and increase in cell growth inhibition (Fig. 7b) by the drug-loaded microspheres were higher than those by the control microspheres (no drugs) (p < 0.05), providing evidence of the cytotoxicity and anti-proliferative effects of the encapsulated drugs.

The stronger effects of the conjugated drugs (PGS-LHRH, PTX-LHRH) are attributed to the conjugation of the LHRH ligand to the anticancer drugs. This is likely to increase the specificity of the binding of the released drugs to the overexpressed LHRH receptors on the MDA-MB-231 cells. Thus, the LHRH-conjugated anticancer drugs are much more effective in targeting the MDA-MB-231 cells than the unconjugated drugs (PGS or PTX).

#### In vitro cytotoxicity and drug uptake

**Figure 8:**
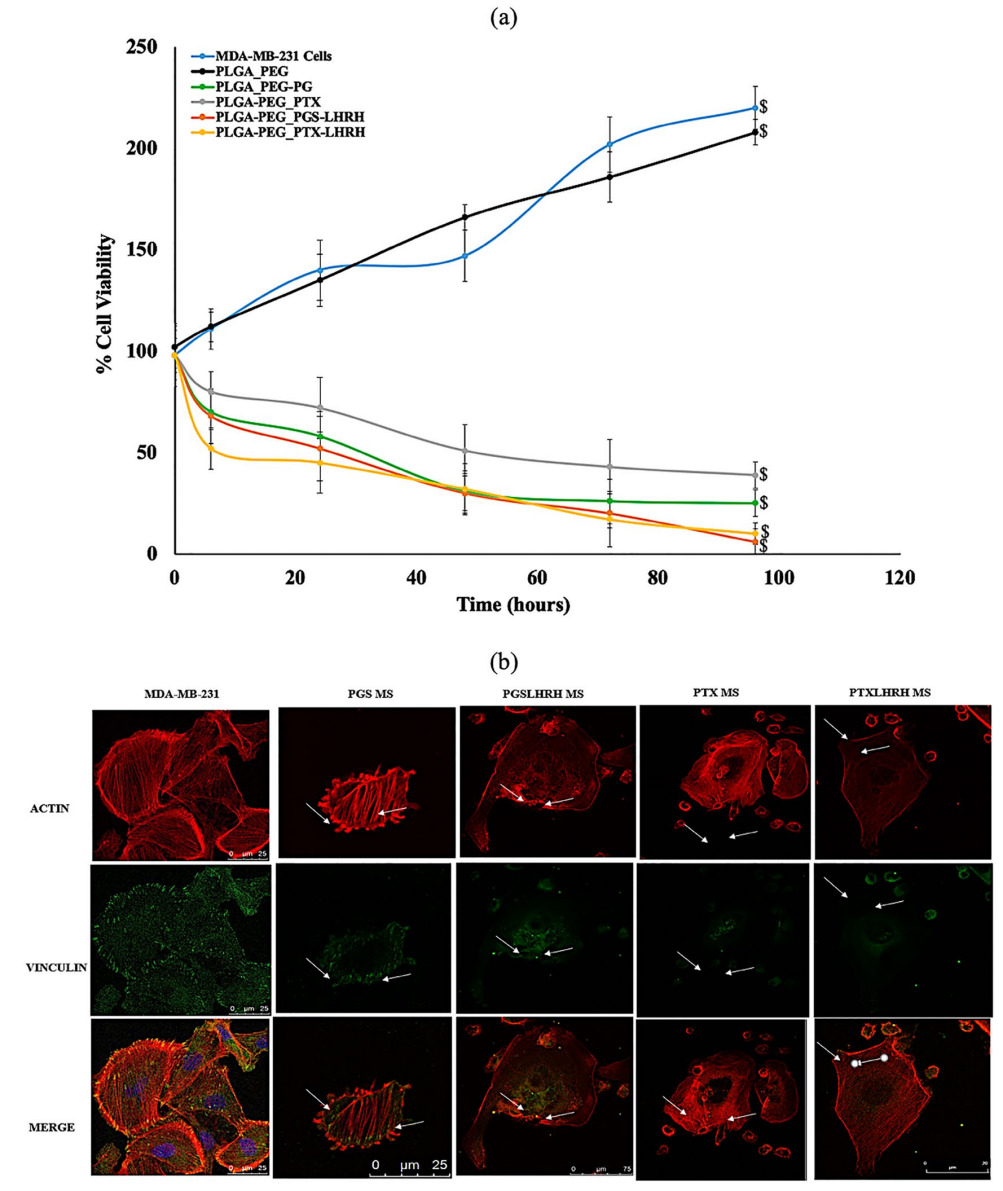
(**a**) Cell viability study of MDA-MB-231 cells showing the effect of the treatment time when incubated with drug-loaded and unloaded PLGA-PEG microspheres after for a period of 240 h with MDA-MB-231 cells acting as a control. (**b**) Representative confocal images of MDA-MB-231 cells after 5 h incubation with respective drug-loaded PLGA-PEG microspheres at 37 °C. Red staining reveals actin-filaments and green staining indicates vinculin. All cells were stained and imaged under the same conditions. White arrows indicate the initiation of cytoskeleton disruption/disintegration (n = 3, ^$^p < 0.05 vs. control).

In this study, we consider the cytotoxicity to be a measure of the percentage of cell growth inhibition. Figure 8a shows the extent to which the addition of the drug-loaded PLGA-PEG microspheres inhibited MDA-MB-231 cell growth after 6, 24, 48, 72 and 96 h of exposure, when compared to the inhibition of untreated cells. Higher cytotoxicity levels (due to drug-treatment) correspond to higher percentages of cell growth inhibition. The results show that cell growth was clearly inhibited by the release of drugs from the drug-loaded PLGA-PEG microspheres (compared to control unloaded PLGA-PEG microspheres).

Furthermore, the cells treated with PLGA-PEG microspheres loaded with conjugated drugs (PGS-LHRH, PTX-LHRH) exhibited higher percentages of cell growth inhibition than their counterparts loaded with unconjugated drugs (PGS, PTX). Hence, the LHRH-conjugated drug-loaded microspheres were more effective at inhibiting cell growth than the unconjugated drug-loaded microspheres. The increased effectiveness of the LHRH-conjugated drugs is attributed to the specific targeting of the LHRH receptors on the MDA-MB-231 cells.

Finally, the Trypan blue dye (TBD) cell count was used to confirm the effects of the drug-loaded PLGA-PEG microsphere treatment on MDA-MB-231 cell viability. An exponential increase in the cell viability/proliferation of the MDA-MB-231 cells (control) was observed throughout the incubation period. In agreement with the Alamar Blue assay results, the viability of the MDA MB 231 cells treated with PLGA-PEG microspheres (loaded with conjugated drug) were significantly reduced, in comparison to MDA-MB-231 cells treated with PLGA-PEG microspheres loaded with unconjugated drugs. This again shows that the conjugated drugs were effective at reducing cell viability than the unconjugated drugs. In summary, the TBD revealed that ~ 95% of the cells were dead (with ~ 5% of viable cells remaining) after 96 h of exposure to targeted encapsulated drug-loaded PLGA-PEG microspheres. The results show a significant difference between the cell viability of encapsulated targeted-drug system (PLGA-PEG_PGSLHRH, PLGA-PEG_PTXLHRH) and PLGA-PEG_PGS, PLGA-PEG_PTX since the p-value calculated is < 0.05.

The network of the cytoskeleton of actin microfilaments, intermediate filaments, and microtubules make up the cytoplasm which controls the mechanical structure and shape of the cell^69^. Hence, the disruption of the spatial organization of the cytoskeleton networks (by pharmacological treatments) can affect the structure and properties of the cell^70,71^. Hence, in this section, changes in the cytoskeleton structure are elucidated following exposure to the release of cancer drugs (PGS, PTX, PGS-LHRH, PTX-LHRH). The resulting effects of the uptake of cancer drugs was elucidated via confocal laser scanning microscopy and are presented in Fig. 8b. Distinctive changes in the cytoskeletal structures were observed after 5 h of exposure to drug release. The changes in the cytoskeletal structure also continue with increasing exposure to the released drugs. This results suggests that the exposure to cancer drugs significantly affects the underlying cytoskeletal structure giving rise to apoptosis and cell death^72–75^.

### In vivo animal studies

**Figure 9:**
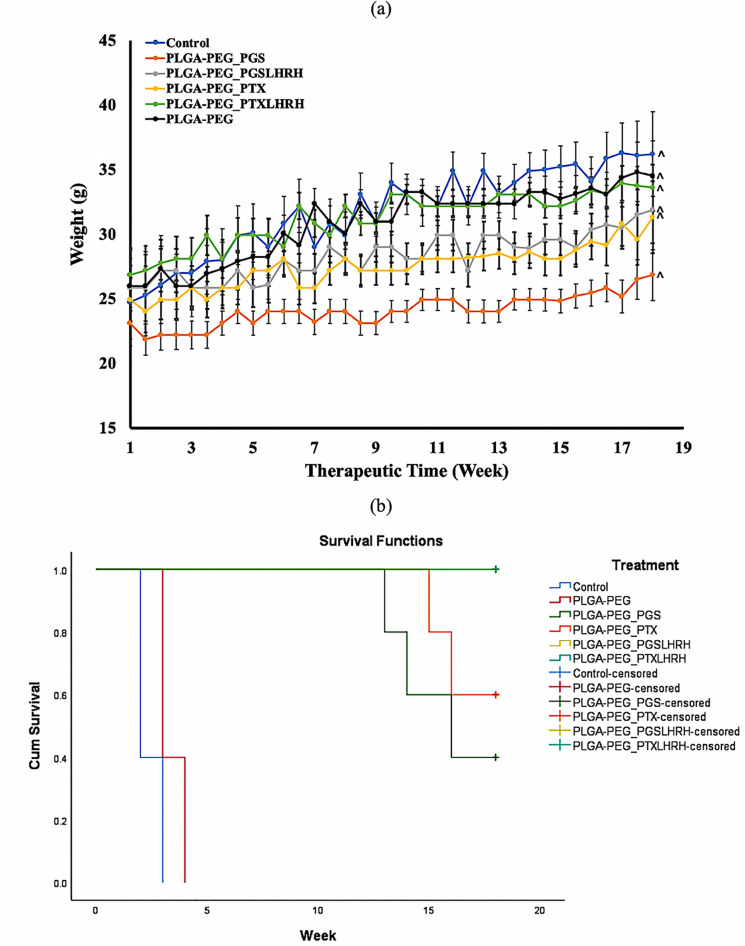
(**a**) Body weight variation of subcutaneous xenograft tumor-bearing mice treated with drug-loaded microparticles in the presence of control (n = 5, ^p < 0.05) (**b**) Kaplan Meier survival curves (N = 30) showing the effect of all treatment groups on the survival rate of mice.

Figure 9a presents the body weights of the mice over the therapeutic period of 18 weeks. Results showed that there were no statistical difference in the growth rate (as a function of weight) of mice treated with drug-loaded microspheres and the control group. It can be concluded that there were no significant changes in the body weight associated with any of the treatment groups as compared to the control group. This implies that the drug-loaded particles used did not create any cytotoxic effects on the general well-being of the treatment group mice during the therapeutic window/time. Although there was an increase in body weight of the treatment groups, this increase is synonymous to those of the control group indicating that there was no noticeable side effects, physiological changes, or drastic decrease in the body weight after the administration of the drugs, compared to the control mice. Consequently, during the therapeutic time, all of the mice studied appeared to be healthy with normal eyes and skin conditions. These results are similar to our previous study in which we found that the concentration of the conjugated drugs used are effective for the treatment of TNBC^31^.

Survival rate for all the treatment groups during the therapeutic duration are shown is presented in the Kaplan–Meier curves as shown in Fig. 9b. A survival rate that describe the recurrence of the treated tumor was observed at week 13, 14, 16 for mice treated with PLGA-PEG_PGS while at week 15 and 16 week we observed a recurrence for mice treated with PLGA-PEG_PTX. ln vivo animal studies results showed that the drug loaded microsphere prolonged the survival of mice and prevented the recurrence time for tumor. However, mice treated with targeted drug-loaded microspheres (PLGA-PEG_PGSLHRH and PLGA-PEG_PTXLHRH) with an overlapping curve show a prolonged survival and limits recurrence compared to the PLGA-PEG_PGS and PLGA-PEG_PTX. Overall, our results reveal that each group treated with drug-loaded microspheres had a higher cumulative survival compared to the cumulative survival noted in the untreated/control groups (p < 0.0001). These results from are in good agreement with the in-vitro cell viability studies.

**Figure 10:**
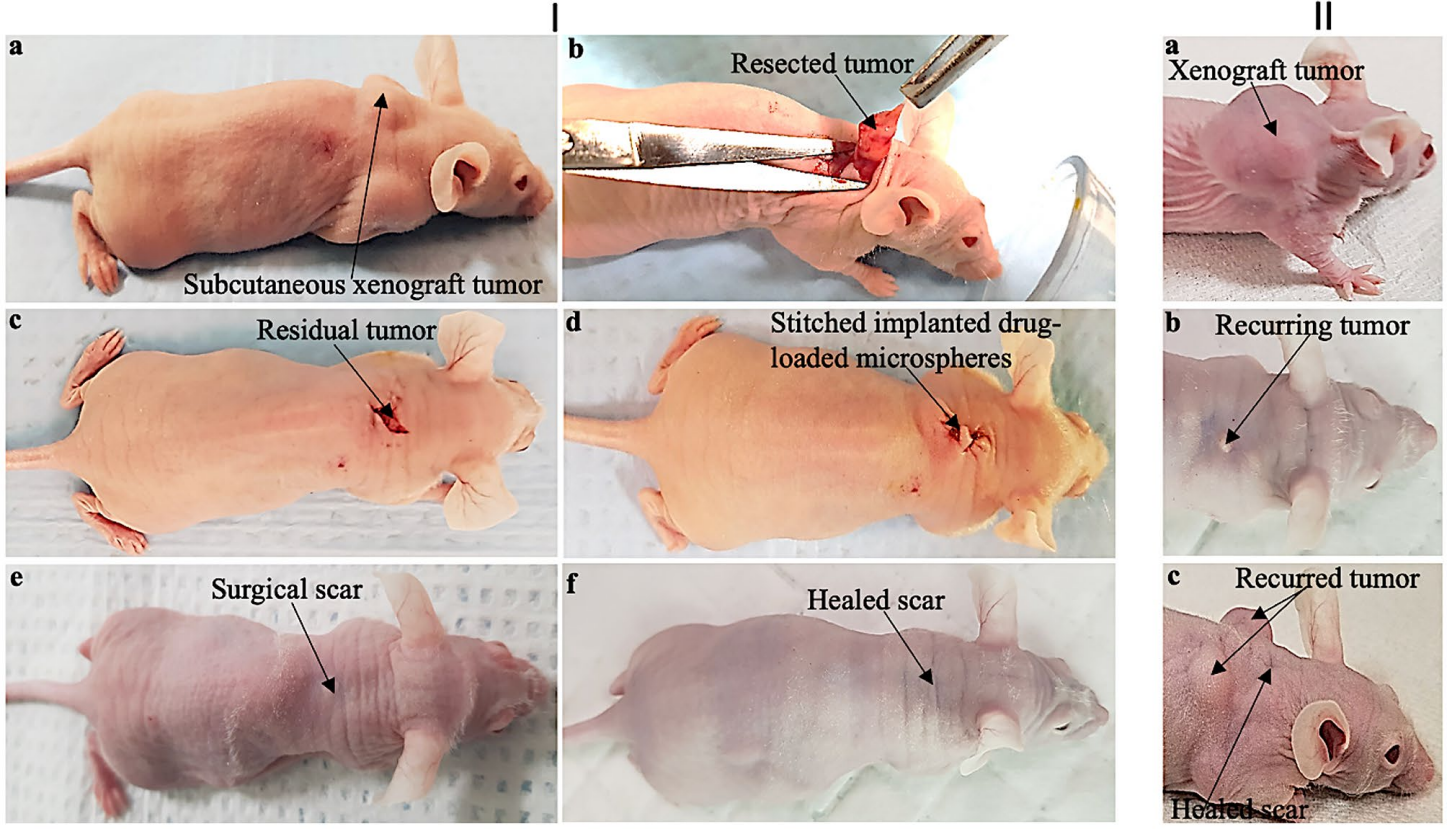
(**I**) Representative photographs showing the steps involved in the treatment of the TNBC tumor with drug-loaded microspheres: (a) subcutaneous xenograft TNBC tumor; (b) surgical tumor removal; (c) residual tumor; (d) stitched residual tumor with implanted drug-loaded microspheres; (e) healing scar 8 weeks after surgery and (f) completely healed mice 18 weeks after surgery and treatment with targeted drug-loaded microspheres (PLGA-PEG_PGSLHRH). (**II**) (a–c) Representative mice treated with non-drug microparticles (PLGA-PEG) with recurred tumor.

The mean tumor volume was 310 ± 14 mm^3^ 28 days after the tumor was induced subcutaneously (Fig. 10Ia). The process of surgery and the outcome of implanted drug-loaded microspheres are shown in Fig. 10I. The representative drug-loaded microspheres (PLGA-PEG_PGS-LHRH) implanted after tumor was removed revealed that there was no local recurrent of tumor after 18 weeks (Fig. 10If). It was observed that for the case of mice implanted with targeted drug-loaded microspheres (PLGA-PEG_PGS-LHRH and PLGA-PEG_PTX-LHRH), there was no recurrence of tumor after drug released from the microspheres for 18 weeks (See representative result in Fig. 10If). These results are also similar for PLGA-PEG_PTX-LHRH. PLGA-PEG_PGS, and PLGA-PEG_PTX.

In general, for the mice treated with targeted drug-loaded microspheres, no significant weight loss or side effects were discussed. However, this groups implanted with positive control microspheres (PLGA-PEG) and the control mice (with no microspheres) exhibited noticeable multiple recurrences of the TNBC tumors (Fig. 10IIa–c). These recurrences are attributed to the incomplete removal of all of the residual tumor and the absence of drug-loaded microspheres (Fig. 10Ic). In contrast, no tumor reoccurrence was observed after the implantation of the targeted TNBC drug (PLGA-PEG_PGS-LHRH). Hence, the drug-loaded microparticles are effective for the prevention of tumor recurrence, following surgical resection of triple negative breast tumor.

**Figure 11:**
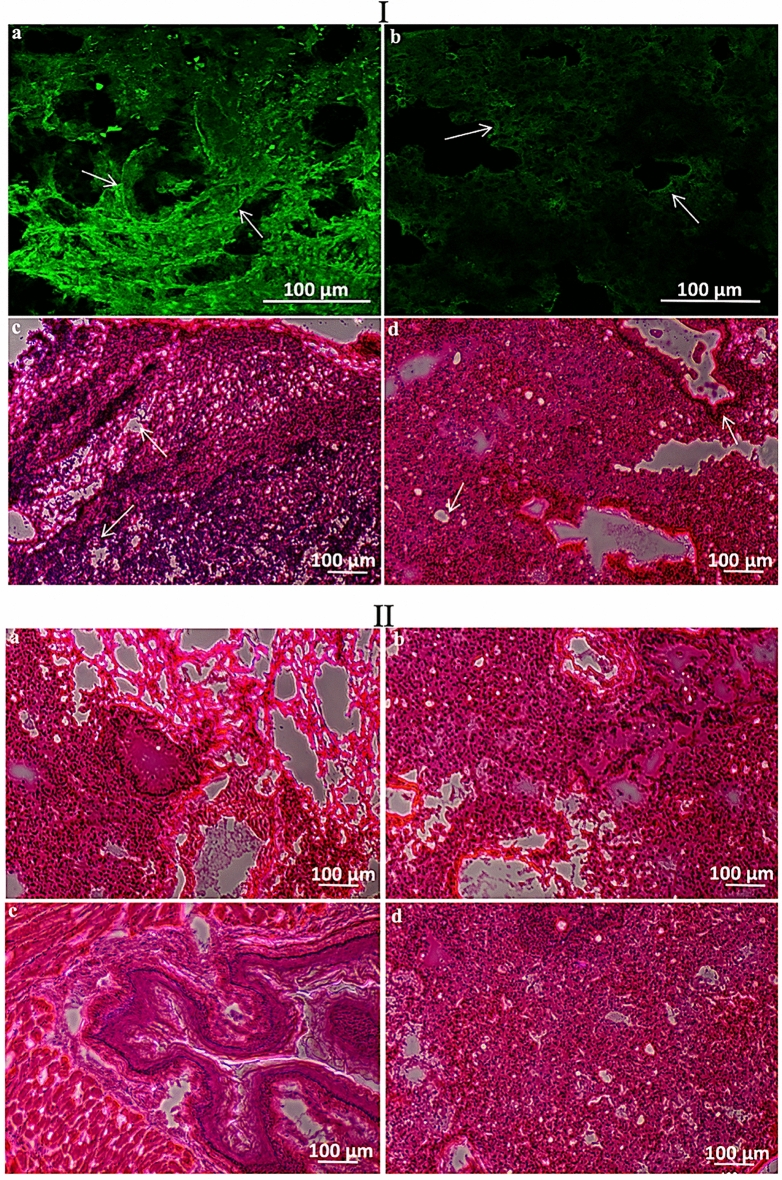
(**I**) Representative immunofluorescence images of LHRH receptors (green stain) expressed on the (a) tumor, and (b) lungs of mice treated with a control microspheres (PLGA-PEG) and their corresponding H&E stain showing metastasis in the (c) tumor and (d) lungs. (**II**) Optical images of mice lungs treated with (a) PLGA-PEG_PTX, (b) PLGA-PEG_PGS, (c) PLGA-PEG_PTX-LHRH, (d) PLGA-PEG_PGS-LHRH microspheres.

Figures 11Ia,Ib) present immunofluorescence (IF) images of LHRH receptors showing the presence of LHRH receptors on the tumor and lungs of the control mice group that was treated with non-drug loaded microparticles. It was also noticed that after 18 weeks of surgery, the source tumor (Fig. 11Ic) showed metastases in the lungs (Fig. 11Id). Figures 11IIa,b show the lungs of mice treated with PTX-loaded PLGA-PEG and PGS-loaded PLGA-PEG microparticles, respectively, while Figs. 11IIc,d show the lungs of mice treated with PTX-LHRH-loaded PLGA-PEG and PGS-LHRH-loaded PLGA-PEG microparticles, respectively. The results clearly show that for the control mice, there was evidence of metastasis in the lungs, due to the presence of multiple metastatic foci or nodules from H&E histological staining. Hence, both IF staining and the H&E analyses of the primary tumors and the metastases in the lungs validated the use of drug-loaded microspheres for the localized drug delivery of PGS-LHRH to tumor sites following surgical removal of the primary tumor.

### Implications

The implications of the above results are very significant. First, uniquely loaded microspheres (of relevant clinical sizes) have been developed for the delivery of targeted cancer drugs (PGS-LHRH, PTX-LHRH) to TNBC cells. The microspheres, which were formulated from a distinct blend of polymers, exhibited bi-phasic release of the anti-cancer drugs. The drug release kinetics are controlled by anomalous (non-Fickian) drug diffusion following the Korseymer–Peppas model at the earlier stages of drug release. This is followed by degradation and membrane erosion, as shown in the SEM degradation images at the later stages of drug elution.

A higher level of burst release was observed for PTX-based drugs than PGS-based drugs. Similar drug release profiles were observed at different temperatures. The thermodynamic studies also confirmed the feasibility of the drug release at different temperatures, while the release kinetics were shown to be controlled by non-Fickian diffusion and polymer degradation, which was confirmed by observations of erosion on the surfaces of the PLGA-PEG microspheres after polymer degradation. These insights into the thermodynamics and kinetics of the drug release create a new dimension that describe processes that are relevant to localized drug release and their mechanisms.

Furthermore, under in vitro conditions, the targeted drugs (LHRH conjugated Prodigiosin/Paclitaxel) were more effective at reducing the viabilities of breast cancer cells (MDA-MB-231) than Prodigiosin/Paclitaxel alone. This suggests that the LHRH enhances the specific targeting of TNBC cells by inhibiting cancer cell growth. Distinct changes in actin cytoskeletal structure and vinculin transmembrane structures were associated with targeted drug release. The in vivo results also suggest that the targeted-drug-loaded microspheres are effective at preventing the locoregional recurrence of TNBC, following surgical resection of triple negative breast tumors.

## Conclusion

The release of targeted and untargeted (PGS and PTX) cancer drugs from physical blends of PLGA and PEG microparticles is presented in this paper. The results show that the blended microcapsules enable the extended release of cancer drugs (PGS, PTX, PGS-LHRH, PTX-LHRH) over periods of 62 days that could greatly facilitate the localized treatment of TNBC. Anomalous drug release was shown to occur by non-Fickian diffusion. This was attributed to polymer degradation and surface erosion phenomena that occur during the later stages of drug elution. The results also show that the elution of targeted drugs (PGS-LHRH and PTX-LHRH) reduces cell viability more than the elution of unconjugated PGS or PTX. Similarly, the in vivo animal experiments on nude mice showed that the targeted drugs (PGS-LHRH and PTX-LHRH) prevent the locoregional recurrence of TNBC, following tumor resection and the implantation of PLGA-PEG microcapsules that elute the targeted drugs. Clearly, the drug-loaded particles extended the survival time of the treated mice post tumor resection compared with the control/untreated mice. This study reveal the potential use of encapsulated targeted drug-loaded PLGA-PEG microspheres for the potential treatment of TNBC tumor after surgery.

## Materials and experimental methods

### Materials

Poly (D,L-lactide-co-glycolide) (PLGA 65:35, viscosity 0.6 dL/g), poly vinyl alcohol (PVA) (98% hydrolyzed, MW = 13,000–23,000), Bovine Serum Albumin (BSA) and 4% paraformaldehyde were obtained from Sigma Aldrich (St. Louis, MO, USA). Polyethylene glycol (PEG) (8 kD), Dichloromethane (DCM) and Phosphate Buffered Saline (PBS) solution that were used for in vitro drug release at pH of 7.4 were purchased from Fisher Scientific (Hampton, NH, USA). Prodigiosin (PGS) were biosynthesized and chemically conjugated with Luteinizing hormone-releasing hormone (LHRH) at the Soboyejo Lab at the Worcester Polytechnique Institute (WPI), Worcester, MA, USA. Paclitaxel was obtained from ThermoFisher Scientific (Walthmam, MA, USA) and was conjugated to LHRH.

Cell culture medium Leibovitz's-15 (L-15), trypsin-ethylenediamine-tetra-acetic acid (Trypsin–EDTA), Fetal Bovine Serum (FBS), penicillin–streptomycin, Alamar Blue Cell Viability Assay, Dulbecco's phosphate-buffered saline (DPBS), vinculin Mouse Monoclonal Antibody, Goat anti-Mouse IgG (H + L) Superclonal Secondary Antibody, Alexa Fluor 488 conjugate, Alexa Fluor 555 Rhodamine Phalloidin, Triton X-100, Trypan Blue Solution (0.4%) were also procured from ThermoFisher Scientific (Walthmam, MA, USA). MDA-MB-231 cell line used in this study was obtained from American Type Culture Collection (ATCC) (Manassas, VA, USA). All of the reagents that were used were of analytical grade, as provided by the suppliers.

### Preparation of drug-loaded PLGA-PEG microspheres

Targeted drug-loaded microspheres (PGS-LHRH-loaded PLGA-PEG and PTX-LHRH-loaded PLGA-PEG blend microspheres) and non-targeted drug-loaded microspheres (PGS-loaded PLGA-PEG and PTX-loaded PLGA-PEG blend microparticles) were prepared, respectively, using the emulsion solvent evaporation technique, described in prior work by Obayemi et al*.*^24,76^. Although, in this study physical blends consisting of PLGA and PEG polymer in the ratio of 1:1 were dissolved in an organic solvent (DCM) to form a primary system. In separate vials, 5 mg/ml drug concentration (PGS or PGS-LHRH or PTX or PTX-LHRH) were prepared and emulsified in a 3% PVA stabilizer. These were then transferred under homogenization to the primary solution.

The resulting drug-polymer mixtures were sonicated to form a homogenous initial oil–water system. The homogeneous emulsion was then transferred dropwise into an aqueous 3% PVA solution (prepared with deionized water). The mixture formed was homogenized with an Ultra Turrax T10 basic homogenizer (Wilmington, NC, USA) that was operated at 30,000 rpm for 5 min. The resulting oil–water emulsion was then stirred with a magnetic stirrer for 3 h to enable the evaporation of the DCM.

The excess amount of PVA in the stirred mixture was removed by washing four times with tap water and centrifuging for 10 min at 4,500 rpm with an Eppendorf Model 5,804 Centrifuge (Hauppauge, NY, USA). The emulsifier/stabilizer and non-incorporated drugs were then washed off, while the drug-encapsulated microparticles were recovered after centrifugation. Finally, the resulting microparticles were lyophilized for 48 h with a VirTis BenchTop Pro freeze dryer (VirTis SP Scientific, NY, USA). The lyophilized microparticles powder were stored at − 20 °C, prior to the material characterization and drug release experiments. PLGA-PEG microparticles (without drugs) were also prepared as controls.

### Drug-loaded microparticles

The hydrodynamic diameters and polydispersity index of the lyophilized drug-loaded and control PLGA-PEG microparticles were analyzed using a Malvern Zetasizer Nano ZS (Zetasizer Nano ZS, Malvern Instrument, Malvern, UK). The morphologies of the microparticles were also characterized using Scanning Electron Microscopy, (SEM) (JEOL 7000F, JEOL Inc. MA, USA). Prior to SEM, the freeze-dried microparticles were mounted initially on double-sided copper tape on an aluminum stub. The resulting particles were then sputter-coated with a 5 nm thick layer of gold. The mean diameter of the microparticles were then analyzed using the ImageJ software package (National Institutes of Health, Bethesda, MD, USA).

Fourier Transform Infrared Spectroscopy (FTIR) (IRSpirit, Shimadzu Corporation, Tokyo, Japan) was used to characterize the physicochemical properties of the drug-loaded PLGA–PEG microparticles. This was used to evaluate the chemical bonds/functional groups that were associated with the drug-loaded and unloaded PLGA-PEG microparticles. The lyophilized samples were scanned at 4 mm/s at a resolution of 2 cm^−1^ over a wavenumber range of 600–3,600 cm^−1^. This was done using the IR solution software package (ver.1.10) (IRSpirit, Shimadzu Corporation, Tokyo, Japan).

Nuclear Magnetic Resonance Spectroscopy (NMR) was also used to study the structure of unloaded and drug-loaded PLGA-PEG microparticles. This was done using a Bruker Advance 400 MHz (Bruker BioSpin Corporation, Billerica, MA, USA). First, 10 mg of PLGA-PEG microparticles were dissolved in 1 ml of chloroform (CDCl_3_). HNMR spectra of drug-loaded and control PLGA-PEG microparticles were obtained and analyzed using Bruker’s TopSpin Software package (ver 3.1) (Bruker Biospin GmbH, Rheinstetten, Germany).

Finally, the thermal properties of the drug-loaded PLGA-PEG microparticles and their control were measured using Thermogravimetric Analysis (TGA) (TG 209 F1 Libra, NETZSCH, Selb, Germany) and Differential Scanning Calorimetry (DSC) (DSC 214 Polyma, NETZSCH, Selb, Germany). This was done to evaluate the possible interactions of the drugs with the polymer blends (PLGA-PEG). TGA thermograms were obtained between 25 and 900 °C with a constant heating rate of 20 K/min under nitrogen gas. This was done using alumina crucibles containing 10 mg of sample.

For the DSC analysis, 10 mg of the freeze-dried drug-loaded and control PLGA-PEG microparticles was weighed, respectively. In each case, samples were sealed in aluminum pans. They were then heated in an inert nitrogen atmosphere with a nitrogen flow rate of 20 ml/min that was subjected to a heating cycle between 20 and 250 °C with an empty reference aluminum pan. The data obtained was then analyzed by NETZSCH Proteus-7.0 software (NETZSCH, Selb, Germany). Similar procedure was followed for DSC analysis of PTX and PGS. This was used to identify the decomposition temperatures, the glass transition temperatures (T_g_) and the melting temperatures (T_m_), respectively.

### In vitro drug release

Sixty-two-day in vitro drug release experiments were performed on PLGA-PEG microparticles that were encapsulated with PGS or PGS-LHRH or PTX or PTX-LHRH. These were carried out at 37 °C, 41 °C and 44 °C in an effort to study the kinetics and thermodynamics of drug release under in vitro conditions. The temperatures were chosen to correspond to the normal human body temperature (37 °C) and hyperthermic temperatures (41 °C and 44 °C).

First, triplicate 10 mg measures of drug-loaded microparticles were suspended separately in 10 ml of PBS of pH 7.4 containing 0.2% Tween 80, using 15 ml screw-capped tubes. The sample tubes were then placed in orbital shakers (Innova 44 Incubator, Console Incubator Shaker, New Brunswick, NJ, USA) rotating at 80 rpm and maintained at temperatures of 37 °C, 41 °C, and 44 °C, respectively. At 24-h intervals, over a period of 62 days, the tubes were centrifuged at 3,000 rpm for 5 min to obtain 1.0 ml of the centrifuged supernatant (known release study samples). 1 ml of freshly prepared-drug free PBS was then used to replace the removed supernatant to conserve the sink conditions. The test samples were then swirled and placed back into the shaker incubator for the continuous release study.

The amount of released drug in each of the supernatant samples (released at 37 °C, 41 °C and 44 °C) was characterized using a UV–Vis spectrophotometer (UV-1900 Shimadzu Corporation, Tokyo, Japan). The wavelength of the UV–Vis spectrophotometer was fixed at a wavelength of 535 nm (PGS and PGS-LHRH) and 229 nm (PTX and PTX-LHRH), respectively, in order to measure the absorbance. A standard curve was used to determine the concentrations of drug (PGS, PGS-LHRH, PTX and PTX-LHRH) released from their respective drug-loaded microparticles^77^.

The drug encapsulation efficiencies of the microspheres were also determined. First, 10 mg of microparticles was dissolved in DCM. The amount of drug encapsulated was then determined with a UV–Vis spectrophotometer (UV-1900 Shimadzu Corporation, Tokyo, Japan) at a fixed maximum wavelength of 535 nm for PGS and PGS-LHRH and 229 nm for PTX and PTX-LHRH, respectively. The amount of drug that was encapsulated into the PLGA-PEG microparticles was then determined from the weight of the initial drug-loaded microparticles and the amount of drug incorporated, using a method developed by Park et al*.*^78^.

The Drug Loading Efficiency and Drug Encapsulation Efficiency (DEE) of drug-loaded PLGA-PEG microparticles was determined from Eqs. (1) and (2), respectively:

1 $$Drug \;encapsulation \;efficency \left( {DLE} \right) = \frac{MD}{{MD + MP}} \times 100$$

2 $$Drug \;encapsulation \;efficency \left( {DEE} \right) = \frac{{M_{X} }}{{M_{Z} }} \times 100$$

where MD is the mass of drug uptake into the microspheres, MP of polymer in the microsphere, M_x_ is the amount of encapsulated drug and M_z_ is the amount of drug used for the preparation of the microparticle.

Since drug release is often enabled by capsule degradation^48^, the degradation of the drug-loaded microparticles was studied after each week of degradation under in vitro conditions. This was done using Scanning Electron Microscopy, (SEM) (JEOL 7000F, JEOL Inc. MA, USA), which was used to characterize the microstructural morphologies of the drug-loaded polymer blend.

### Modeling

#### Kinetics modeling

The drug release kinetics of drug-loaded PLGA-EG microparticles were determined by fitting the release data to Zeroth order kinetics, First Order Kinetics, Higuchi Model and Korsmeyer–Peppas Model. We initially used Zeroth order kinetics to describes the release from the drug-loaded microspheres in which the release rate is independent of concentration^79^. Hence, the plot of % Cumulative Drug Release (CDR) versus time was obtained based Eq. (3) below:

3 $$Q_{t} = Q_{O} + K_{0} .t$$

where Q_t_ is the cumulative amount of drug released in time ‘t’ (release occurs rapidly after drug dissolves), Q_0_ is the initial amount of drug in the solution and K_0_ is the zeroth order release constant and ‘t’ is time in hours.

In the case of first order kinetics, our release rate was shown to depend on concentration^80^. A plot of log of % cumulative drug release (CDR) versus time that gives a straight line was plotted based on Eq. (4):

4 $$\log Q_{t} = \log Q_{0} + {\raise0.7ex\hbox{${Kt}$} \!\mathord{\left/ {\vphantom {{Kt} {2.303}}}\right.\kern-\nulldelimiterspace} \!\lower0.7ex\hbox{${2.303}$}}$$

where Q_t_ is the cumulative amount of drug release in time ‘t’, Q_0_ is the initial amount of drug in the solution, K is the first order release constant, and ‘t’ is time. First order kinetics is often observed during the dissolution of water-soluble drugs in porous matrices ^81^.

Furthermore, the Higuchi model was used to characterize the release of the drugs incorporated into polymer matrices^82,83^. Typically, the Higuchi model describes the drug release from insoluble matrix as a square root of time, based on Fick’s first law^57,58^. A plot of % Cumulative Drug Release (CDR) versus the square root of time $\left( {\sqrt t } \right)$ as shown by Eq. (5) was used to describe the kinetics of drug release.

5 $$Q_{t} = K_{H} .t^{{{\raise0.7ex\hbox{$1$} \!\mathord{\left/ {\vphantom {1 2}}\right.\kern-\nulldelimiterspace} \!\lower0.7ex\hbox{$2$}}}}$$

where *Q*_*t*_ is the cumulative amount of drug released at time (t), K_H_ is Higuchi constant and ‘t’ is time.

Finally, the Korsmeyer-Peppas (K-P) model was also used to explore the drug release kinetics from the polymeric matrix systems. For K-P drug release, a plot of log $\frac{{M_{t} }}{{M_{\infty } }}$ versus log t was plotted where ‘n’ represents the slope of the line, which corresponds to the underlying mechanism of drug release. The diffusion exponent (*n* value) of Korsmeyer-Peppas model was then used to identify the different drug release mechanism. For example, *n* < 0.45 corresponds to a Fickian diffusion mechanism, while 0.45 < *n* < 0.89 corresponds to non-Fickian transport, *n* = 0.89 corresponds to Case II (relaxational) transport, while *n* > 0.89 corresponds to super case II transport^47,80,82^. The K-P model is given by (6):

6 $$\frac{{M_{t} }}{{M_{\infty } }} = Kt^{n}$$

where $\frac{{M_{t} }}{{M_{\infty } }}$ is a fraction of drug released after time ‘t’, ‘K’ is the kinetic constant, n is the release exponent, and ‘t’ is time. In most cases, the K-P model is only applicable to the first 60% of drug release ^80,81^.

#### Thermodynamics of in vitro drug release

The drug release studies were used to obtain the Gibbs free energy (Δ*G*), the enthalpy (Δ*H*), and the entropy (Δ*S*) changes associated with drug release from the drug-loaded PLGA-PEG microparticles at different temperatures^84,85^. The values of Δ*G*, Δ*H* and Δ*S* obtained were then used to explain the thermodynamic properties and the spontaneity of the underlying drug release processes from the drug-loaded microspheres.

Initially, the experimental data obtained from our drug release experiments (at different temperatures) were used to estimate the activation energy (*E*_a_). This is done using the Arrhenius Eq. (8). The underlying thermodynamical mechanisms were then elucidated from Eqs. (7) and (8). These give:

7 $$K_{t} = D_{f} e^{{\frac{{E_{a} }}{RT}}}$$

and

8 $$lnK_{t} = lnD_{f} - \frac{{E_{a} }}{R} \frac{1}{T}$$

where *R* is the universal gas constant (8.314 J mol^−1^ K^−1^), *K*_t_ is the thermodynamic equilibrium constant, *T* is given as the absolute temperature (K), *E*_a_ is the activation energy, D_f_ is the pre-exponential factor and K_t_ is the thermodynamic equilibrium constant. The activation energy, *E*_a_ (kJ mol^−1^), was estimated from a Van Hoff plot of lnK_t_ versus 1/*T*. Hence, the slope of the plot gives $- \frac{{E_{a} }}{R}$. The Eyring expression for *K*_t_ gives (9):

9 $$\ln \frac{{K_{t} }}{T} = - \frac{\Delta H}{R}\frac{1}{T} + \ln \frac{{K_{B} }}{h} + \frac{\Delta S}{R}$$

In cases in which the plot of $$\ln K_{t}$$ versus $${\raise0.7ex\hbox{$1$} \!\mathord{\left/ {\vphantom {1 T}}\right.\kern-\nulldelimiterspace} \!\lower0.7ex\hbox{$T$}}$$ is linear, then the underlying enthalpy Δ*H* (slope) and entropy Δ*S* (intercept) can be determined, respectively, from the slopes and intercepts of the plots^84^. Hence, the slope ‘m’ is given as $$- \frac{\Delta H}{R}$$ and the intercept ‘c’ is given by $$\ln \frac{{K_{B} }}{h} + \frac{{{\Delta }S}}{R}$$ where Δ*H* is the enthalpy change, Δ*S* is the entropy change, K_B_ is the Boltzmann constant (1.38065 m^2^ kg s^−2^ k^−1^), and h is the Planck’s constant (6.626 × 10^−34^ J s). Finally, the changes in the free energy $$\Delta G$$ can be obtained by substituting the calculated values of Δ*H* and Δ*S* into Eq. (10) at a given temperature, T.

Finally, the Gibbs free energy change is given by (10):

10 $$\Delta G = \Delta H - T\Delta S$$

where $\Delta S$ is the entropy change, $\Delta H$ is the enthalpy change and $\Delta G$ is Gibbs free energy change.

### Cell culture experiments

The MDA-MB-231 breast cancer cells were cultured in Leibovitz's 15 (L-15) medium, supplemented with 10% FBS and penicillin/streptomycin (50 U/ml penicillin; 50 μg/ml streptomycin). This complete cell culture medium containing L-15 and other supplements (10% FBS and 2% penicillin/streptomycin) is referred to as L-15^+^.

#### In vitro cell viability and cytotoxicity

In vitro cell viability and cytotoxicity studies were performed using the Alamar Blue Cell Assay as described in our recent studies^31^. This was used to explore the possible effects of drug-induced toxicity on triple negative breast cancer (MDA-MB-231) cells. 10^4^ cells/well were seeded in 24-well plates (n = 4) in L-15^+^ culture medium^31^. Furthermore, three hours after cell attachment, the culture medium was replaced with 1 ml of culture medium containing 0.5 mg/ml drug-loaded PLGA-PEG microparticles.

Cell viability was monitored at durations of 0, 6, 24, 48 72 and 96 h after drug-loaded microparticle addition. At each of these time points, the culture medium (L-15^+^) was replaced with 1 ml of culture medium (L-15^+^) containing 10% alamar blue solution. The resulting cells in the 24 well-plates were then incubated in a humidified incubator at 37 °C for 3 h. 100 μl aliquots were transferred into duplicate wells of a black opaque 96-well plate (Thermo Fisher Scientific, Waltham, MA) for fluorescence intensities measurement at 544 nm excitation and 590 nm emission using a 1420 Victor3 multilabel plate reader (Perkin Elmer, Waltham, MA)^31^. All of the experiments were repeated thrice.

The percentage of alamar blue reduction and the percentage of cell growth inhibition were determined from Eq. (11) and (12)^31^:

11 $$\% Reduction = \frac{{FI_{sample} - FI_{10\% AB} }}{{FI_{100\% R} - FI_{10\% AB} }} \times 100$$

12 $$\% Growth \;inhibition = \left( {1 - \frac{{FI_{sample} }}{{FI_{cells} }}} \right) \times 100$$

where $FI_{sample}$ is the fluorescence intensity of the samples$,{ }FI_{10\% AB}$ is the fluorescence intensity of 10% Alamar Blue reagent (negative control)$, FI_{100\% R}$ is the fluorescence intensity of 100% reduced Alamar Blue (positive control)${\text{ and }}FI_{cells}$ is the fluorescence intensity of untreated cells^31^.

The loss of cell viability was characterized using a dye exclusion assay. This works based on the concept that viable cells do not take up impermeable dyes (like Trypan Blue), while dead cells are permeable and take up the dye because their membranes lose their integrity. Hence, we adopted previous method reported in our prior work^24^. In this work Trypan Blue Dye (TBD) staining was used to quantify the loss of cell viability. This utilized a 0.4% solution of TBD in buffered isotonic salt solution with a pH of 7.3. 0.1 ml of TBD stock solution was added to 1 ml of cells, mixed gently and incubated at 25 °C for 1 min. A hemocytometer was then used to count the number of blue staining cells, and the total number of cells under an optical microscope (Nikon TS100, Nikon Instruments Inc., Melville, New York, USA) that was operated at low magnification^24^.

13 $$\% Viable\; cells \left( {VC} \right) = 1 - \left( {Number\; of\; blue\; cells \div Number\; of \;total \;cells} \right) \times 100$$

#### Cellular drug uptake

MDA-MB-231 cells were seeded on coverslips (CELLTREAT Scientific Products, Pepperell, MA, USA) in 12-well plates using 1 ml growth medium (L-15^+^). The cells were then incubated in a humidified incubator at 37 °C until cells were about 70% confluent. Post attachment, the cells were incubated with 1 ml of 0.1 mg/ml drug-loaded microspheres dissolved in growth medium (L-15^+^). After 5 h, the cells were washed twice with 5% (v/v) Dulbecco's phosphate-buffered saline (DPBS) (Washing solvent). After washing, the cells were then fixed with 4% paraformaldehyde for 12 min, before rinsing thrice with 5% (v/v) DPBS. 0.1% Triton X-100 was added for 10 min to permeabilize the cells^86^. This was then blocked with 1% BSA for 1 h at room temperature (25 °C). The BSA-treated ECM were then rinsed thrice with the 5% (v/v) DPBS, before labeling with vinculin Mouse Monoclonal Antibody at 2 µg/ml and incubating for 3 h at room temperature (25 °C).

The washing solvent was used to rinse the resulting samples, which were then labeled with Goat anti-Mouse IgG (H + L) Superclonal Secondary Antibody, Alexa Fluor 488 conjugate for 45 min at room temperature. F-actin was stained with Alexa Fluor 555 Rhodamine Phalloidin for 30 min. The coverslips were then mounted on glass slides and sealed. The cells were visualized with HEPES buffer (pH 8) using HCX PL APO CS 40X 1.25 oil objective in Leica SP5 Point Scanning Confocal Microscope (Buffalo Grove, IL, USA) and representative images were obtained.

### In vivo studies

In vivo animal studies similar to our recent studies^31^ were carried in this work using thirty 3-week old healthy immunocompromised female athymic nude-Foxn1nu mice. These mice were purchased from Envigo (South Easton, MA, USA) and have a weight of ~ 16 g. These mice were kept in the vivarium (to acclimatize) until they are 4-weeks old. They were then used in in vivo studies to explore the extent to which encapsulated localized and targeted drug delivery systems can be used to prevent the breast tumor regrowth or locoregional recurrence, following surgical resection^31^.

All the animal procedures described in this work were performed in accordance with the approved animal guidelines by the Worcester Polytechnic Institute (WPI), Institutional Animal Care and Use Committee (WPI IACUC) with approval number #A3277-01. The mice were also maintained in accordance with the approved IACUC protocol and were provided with autoclaved standard diet^31^. All the experimental protocols in these studies were performed under an approved ethical procedure and guidelines provided by the Worcester Polytechnic Institute IACUC. The sample group are based on the agent that are implanted into the mice for the treatment. The number of mice per this sample group (n) was determine to be n = 5 based on power law and from our prior work. The thirty mice were randomly divided into six groups of five mice each. Each of this group was exposed to one of the following: (PLGA-PEG_PGS, PLGA-PEG_PGS-LHRH, PLGA-PEG_PTX, PLGA-PEG_PTX-LHRH), positive control (PLGA-PEG) and control group (without microsphere).

When the mice in each study group were 4-weeks-old, we induced interscapular subcutaneous TNBC tumors via the subcutaneous injection of 5.0 × 10^6^ MDA-MB-231 cells that were harvested from monolayer in vitro cell cultures^31^. Subcutaneous tumors were allowed to grow for over 4 weeks until they were large enough to enable tumor surgery and microsphere implantation (28 days after tumor induction). The expected size of the induced subcutaneous xenograft tumor after 28 days of induction is 300 ± 21 mm^3^^31^. The tumor formation was investigated by palpation, which was measured on a daily basis with digital calipers. During this period, the mice were monitored for changes in weight, abnormalities and infections. For baseline evaluation, control mice (without microspheres) were also monitored for comparisons with the mice injected with drug-loaded microspheres.

Tumor volume was calculated from the following formula^87,88^:

14 $$Tumor = a \times {\raise0.7ex\hbox{${b^{2} }$} \!\mathord{\left/ {\vphantom {{b^{2} } {2 }}}\right.\kern-\nulldelimiterspace} \!\lower0.7ex\hbox{${2 }$}}$$

where a and b are the respective longest and shortest diameters of the tumors that were measured using a digital Vernier caliper.

Surgical removal of ~ 90% of the tumor was performed randomly on each group member using the recommended anesthesia and pain suppressant. In each case, 200 mg/ml of PLGA-PEG_PGS, PLGA-PEG_PGS-LHRH, PLGA-PEG_PTX, PLGA-PEG_PTX-LHRH, positive controls (PLGA-PEG) and control were implanted locally at the location where the source resected tumor was removed^31^. The statistical rationale for each treatment group was based on power law and from our prior work^31^. Within each group, localized cancer drug release was monitored for the period of 18 weeks. The body weight of each mice was monitored and measured every 3 days up to 126 days to check for any possible weight loss/gain, physiological changes, toxicity to the drugs, and well-being of the mice for the different treatment groups. This was done to check for possible tumor regrowth^31^. In a similar fashion, after the 18 weeks of study, the mice were euthanized and their tumors and lungs were then excised^31^. This was followed by cryo-preservation to check for any toxicity and metastasis.

Following weight analysis, we compared the survival rate of the various treatment groups as a function of recurrence of the TNBC tumor. Survival study of mice was done post-surgical removal of tumor and during treatment period. The mice were observed for 18 weeks post treatment for signs of cancer recurrence, if any. This was to allow enough time for recurrence. Thirty female nude mice were randomly divided into the following six groups (n = 6): Control, PLGA-PEG, PLGA-PEG_PGS, PLGA-PEG_PGSLHRH, PLGA-PEG_PTX, PLGA-PEG_PTXLHRH, PLGA-PEG. Survival curves were made using Kaplan–Meier plots, and the statistical difference was evaluated using the log-rank test in SPSS. The mice in this study were euthanized when reoccurrence were observed. At the end of week 18, the surviving mice were also euthanized.

### Histopathological study and immunofluorescence staining

The histopathology of the lungs, and in some cases regrowth/reoccurred tumor were evaluated. The samples that were used for the histological examination of the lungs were sectioned into 5 μm thicknesses along the longitudinal axis using similar technique from our recent studies^86^. They were then placed on a glass slide. First, the slides were hydrated by passing them through 100, 90 and 70% of alcohol baths. The hydrated samples (on the slides) were then stained with hematoxylin and eosin (H&E). The stained slides were finally examined using light microscopy (with a 20 × objective lens) in a model TS100F Nikon microscope (Nikon Instruments Inc., Melville, NY, USA) that was coupled to a DS-Fi3 C mount that was attached to a Nikon camera.

Receptor staining via immunofluorescence (IF) staining was used to characterize the overexpressed LHRH receptors on the TNBC tumor and organs. This was crucial to show evidence of regrowth or the presence of metastasis in the organs using the IF staining method as described in prior work^31^. Optimum cutting temperature (OCT) compound-Embedded frozen tumor/tissue were processed in a cryostat (Leica CM3050 S Research Cryostat, Leica Biosystems Inc., Buffalo Grove, IL, USA)^31^. The stained samples were then imaged at a magnification of 40 × in a Leica TCS SP5 Spectral Confocal microscope that was coupled to an Inverted Leica DMI 6000 CS fluorescence microscope (Leica, Buffalo Grove, IL, USA)^31^.

### Statistical analysis

The results are reported as mean ± standard deviation for n = 3 (unless otherwise stated). In the in vitro study of drug release, cell viability studies as well as the in vivo study of the effects of drug release, statistical differences between the treatment groups were analyzed using one-way ANOVA. Differences in in vitro cell viabilities between the different treatment groups at different durations were analyzed using two-way ANOVA with post hoc Tukey HSD multiple comparisons tests using IBM SPSS Statistics 25 package. The differences were considered to be significant when the p-value was < 0.05.
